# Photoelectrochemical studies of DNA-tagged biomolecules on Au and Au/Ni/Au multilayer nanowires

**DOI:** 10.1186/1556-276X-6-535

**Published:** 2011-09-30

**Authors:** Viswanathan Swaminathan, Hwi Fen Liew, Wen Siang Lew, Lanying Hu, Anh Tuan Phan

**Affiliations:** 1 School of Physical and Mathematical Sciences, Nanyang Technological University, 21 Nanyang Link, 637371, Singapore

## Abstract

The use of nanowires (NWs) for labeling, sensing, and sorting is the basis of detecting biomolecules attached on NWs by optical and magnetic properties. In spite of many advantages, the use of biomolecules-attached NWs sensing by photoelectrochemical (PEC) study is almost non-existent. In this article, the PEC study of dye-attached single-stranded DNA on Au NWs and Au-Ni-Au multilayer NWs prepared by pulse electrodeposition are investigated. Owing to quantum-quenching effect, the multilayer Au NWs exhibit low optical absorbance when compared with Au NWs. The tagged Au NWs show good fluorescence (emission) at 570 nm, indicating significant improvement in the reflectivity. Optimum results obtained for tagged Au NWs attached on functionalized carbon electrodes and its PEC behavior is also presented. A twofold enhancement in photocurrent is observed with an average dark current of 10 μA for Au NWs coated on functionalized sensing electrode. The importance of these PEC and optical studies provides an inexpensive and facile processing platform for Au NWs that may be suitable for biolabeling applications.

## Introduction

Gold (Au) nanostructures have paved the way to map out a novel platform for designing nano biobarcode for a wide range of biosensing applications [1]. Au nanomaterials, such as nanoparticles, nanowires (NWs), and nanorods, are the widely studied materials which have great demand in the scientific community [2,3]. Interestingly, they offer a number of properties that make them suitable for use in biological applications, such as biosensing [4], biosorting [5], and biolabelling [6]. The structure and composition in multilayered gold NWs will escalate the development of bio-nanotechnology when compared with nanoparticles [7]. In particular, 1D Au nanostructures have a strong optical property that can be tuned by controlling the wire length and diameter of the NWs and multilayer NWs [8]. Moreover, the optical absorption coefficient of gold NWs is much higher than those of gold nanoparticles [9-11]. The fabricated NWs are tagged with various DNA libraries, antibodies, or antigens that can be used for sensing or labeling at a time of different biological assays through direct chemical reactions [12].

A suitable synthesis technique is needed to control the shape and size of the NWs to improve the biocompatibility for biosensing applications. The most direct approach of controlled synthesis of NWs is produced by electrochemical routes [13]. High aspect ratio NWs have more intense reflection and scattering properties; dominated by the polarization-dependent plasmon resonance between the metallic layers rather than by the bulk metallic reflectance [12]. The identification of tagged biomolecules on the surface of nanomaterials can be encoded and easily read out through optical microscope [14]. The optical properties of Au or Au stripes nanostructures [15], optical quenching [16], and the NW aggregation [17] have widely been reported, but the understanding of surface plasmon for multilayer NWs is still to be explored. Hence, it is important to study the shape of multilayer NWs that affects the surface plasmon [18,19], which is the key area to tune the optical properties of biobarcode in multiplex biolabeling applications.

Photoelectrochemical (PEC) measurements have been well exploited for photovoltaic applications, but the literature is scarce on the detection of biomolecules using this approach. PEC is simple and offers an alternative method of detecting biomolecules through molecular binding on a working electrode by electrochemical route. Thus, we study the PCE properties of tagged Au nanostructures. In this article, we describe the effect of surface plasmon and the variation of luminescence properties on shape-controlled Au nanostructures that tagged with thiolated cy3-dye attached on DNA. We also study the PEC properties of dye with DNA-tagged Au and multilayer NWs coated on functionalized carbon electrode.

## Experimental procedures

**Figure 1:**
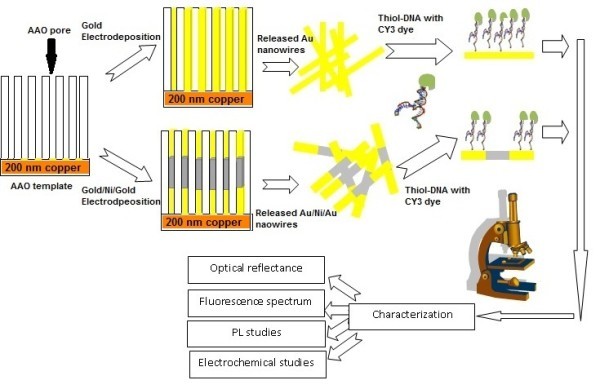
**Schematic illustration of the synthesis of Au NWs and Au/Ni/Au multilayer NWs using pulse electrochemical deposition techniques**.

Figure 1 depicts the preparation of Au NWs and multilayer (Au/Ni/Au) NWs. The starting reactants were of high-purity ammonium gold sulfite electroplating solution (Metalor, 99.99%), nickel sulfate hexahyrate, and boric acid (Fisher Scientific) and sodium citrate (Sigma Aldrich) for the preparation of Au NWs and multilayer NWs. Deoxyribonucleotide triphosphate (dNTP), fluorophores Cy3-dye, and pH 7.4 phosphate buffer solution (PBS) XL (Invitrogen) were used for tagging process. 1-Butyl-3-methylimidazolium hexafluorophosphate (BMIM-PF_6_) (Sigma Aldrich) ionic liquid served as catalysis for PEC measurements. Anodic aluminum oxide (AAO) template (Anodisc 13, Whatman) of high purity and uniform pore density, with an average pore diameter of 200 nm and a template thickness of 60 μm, was employed for pulse electrodeposition [6]. A 200-nm thick copper layer was thermally evaporated onto one side of the AAO template which acted as the working electrode for the pulse electrochemical deposition. The pulse electrodeposition was carried out on the AAO nanopores, using a standard three-electrode potentiostat system (PAR-Verstat-3). A saturated calomel electrode (SCE) was used as the reference electrode, the Cu-coated AAO as cathode, and a platinum mesh was used as the counter electrode. The preparation of gold and nickel layers was produced from 0.1 M of the ammonium gold sulfite electroplating solution and the 0.5 M of nickel salts; and the brightness of the Ni layer was enhanced by adding 0.1 M of boric acid. Multilayer NWs were prepared using separate deposition electrolytes. Under the potentiostatic condition, the deposition potential of the gold and Ni layers was plated at -1.0 V versus SCE, and -1.5 V versus SCE, respectively. Three metallic layers of Au/Ni/Au multilayer NW deposition were carried out at a three-step process. Deposition time and pulse period are two key parameters that can be used to control the NWs lengths. Au NWs and multilayer NWs were separated by etching out the AAO using 3 M sodium hydroxide (NaOH) solution and decanting the dissolved alumina. The released NWs were dispersed in isopropanol alcohol (IPA) and a drop of NWs-IPA mixture was coated on Si substrate for further analysis.

Field emission scanning electron microscope (JSM-6335 FESEM) was employed to study the morphology of Au NWs and multilayer NWs. A bright-field reflectance images were acquired using an inverted microscope (Olympus BX 51,175 W ozone-free He lamp), equipped with a color digital video camera (Sony Exwave HAD-12 megapixel). All reflectance images were taken at 540 nm, which is the wavelength that gives the optimum reflectance area of the Au NWs. A confocal Raman system (WITEC CRM-200) with a processing time of 0.5 s was used to measure the photoluminescence (PL) spectrum of Au NWs and multilayer NWs.

Au NWs and multilayer NWs (150 μL) were first incubated with dNTP (0.2 μL, 10 mM) for 15 min. Then, 300 μL buffer containing NaCl (50 mM) and sodium phosphate (5 mM) was added into the mixture. The volume was reduced to 150 μL by vacuum centrifugation over 4-5 h at 45°C to gradually increase salt concentration which is critical to maintain a stable colloid solution. Then, thiol-DNA was introduced in, followed by heating at 55°C for 3 h. Subsequently, the particles were washed through centrifugation to remove unbound oligonucleotides. Fluorescence of the tagged DNA on gold was accomplished by means of a fluorophores-Cy3-dye (green emission) which was covalently attached to the oligonucleotides used in the sequence of (5'-3'): (5ThioMC6-D/TTT TTT TTT TCC CTA ACC CTA ACC CTA ACC CTT/3Cy3Sp).

PEC measurements were carried out using a three-electrode electrochemical cell and a light source of 200lumens LED (Fenix PP). The resistance of the screen printed electrode was 50 ± 10 Ω. To improve the conductivity of the electrode, 2 μL of BMIM-PF_6 _ionic liquid was coated on the screen-printed carbon surface. The significance of the ionic liquid is that it can improve the conductivity, resulting in low ohmic losses and high rate of mass transfer. Au NWs were then drop-cast on the functionalized screen-printed electrodes. Before electrochemical detection of biomolecules, the dried electrodes were rinsed with pH of 7.4 PBS for further analysis. A three-electrode setup consisting of the functionalized electrode as photo cathode, SCE as reference, and the platinum electrode as anode were used to measure photocurrent upon light irradiation. 20 mL of PBS was used as electrolyte; photocurrent was then recorded as a function of light irradiation. PEC measurements were taken for raw electrode, dark, and light current measurements for the surface-modified photo cathode.

## Results and discussion

**Figure 2:**
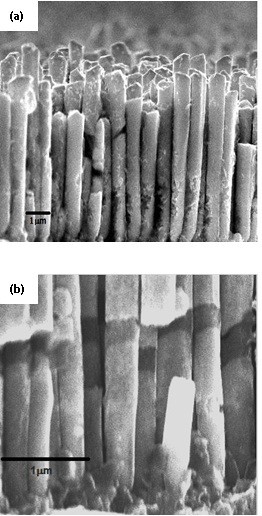
**SEM micrographs of as-prepared (a) Au NWs and (b) Au/Ni/Au multilayer NWs**.

Figure 2a shows a typical FESEM image of pulse electrodeposited Au NWs and multilayer NWs. The diameters of the Au NWs are in the range of approximately 300 ± 30 nm. The observed wire length was inhomogeneous possibly because of the difference in the thickness of the base substrate layer at each pore, or hydrogen uptake which could influence the base crystal nucleation. The Au NWs are continuous and have an average length of 6 μm. The multilayer Au/Ni/Au NWs show a distinct contrast between gold and Ni layers (Figure 2b) and an average length of about 6 μm. The presence of the Ni layer is useful for tagging of multiple biomolecules, magnetic controlled bio sorting in microfluidics device, and easy to handle after washing using a permanent magnet.

**Figure 3:**
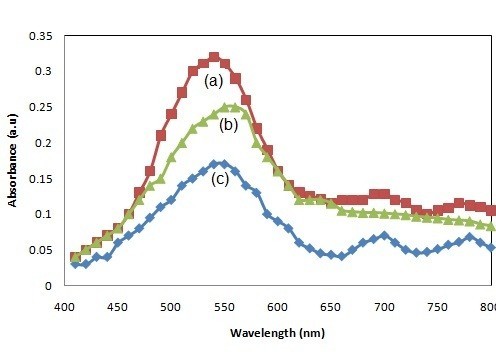
**UV-Vis absorption spectra of as-prepared (a) Au NWs in IPA and (b) Au/Ni/Au NWs in IPA (c) functionalised Au NWs dispersed in PBS solution**.

Figure 3 depicts the optical absorbance spectrum of the as-prepared and dye-attached DNA-tagged Au and multilayer NWs. From the optical investigation, the as-prepared NWs and multilayer NWs were suspended in IPA and water; its absorption spectra for different samples were recorded. The surface plasmon band of metal particles is most responsible for the degree of aggregation and also sensitive to size and shape of the nanostructures. Major absorption peak was recorded at 540 nm as prepared Au NWs (Figure 3a). Furthermore, it was assigned to an interaction with a surface plasmon polariton mode [20]. The Ni layer in multilayer structure showed no reflection in the visible spectrum, when compared with Au layers (Figure 3b). From Figure 3c, the maximum absorption peak shifted to 550 nm which is because of the dye-attached DNA on Au NWs.

The Au NWs were dispersed in different solvents: water and IPA. Owing to different refractive index of the solvents, the reflected intensity of the plasmon band varies significantly with respect to the solvents. Hence, the optical absorbance of Au NWs in IPA is stronger than that in water. It is anticipated that the surface plasmon was dependent on the shape of the particles, the nature of the dispersing solvent, and the aggregation of nanomaterials [14]. Therefore, the maximum optical absorption was observed for Au NWs, particularly dispersed in IPA (Figure 3a). The absorption behavior was different, even though similar size of templates was used for the synthesis of Au NWs and multilayer NWs. The optical absorbance was lower in multilayer NWs because of the amount of wire aggregation and the force of attraction between the wires as Ni is a ferromagnetic material. An enhanced intensity of the plasmon with less aggregation can be obtained when suitable dispersing solvent was used. In Au NWs, there is a minor shift in the absorbance band toward longer wavelengths at 660 and 770 nm, which can be attributed to shape the NWs and coupling between the Au NWs aggregation [21].

**Figure 4:**
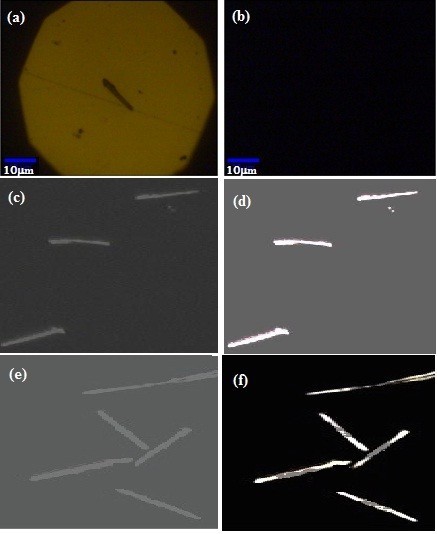
**Dark field optical reflectance and corresponding fluorescence images of released Au NWs**. (a) Bright-field reflectance of Au NWs without tagging; (b) fluorescence image of Au NWs without tagging; (c) and (e) dark field images of Au and Au/Ni/Au NWs with tagging; (d) and (f) fluorescence images of Au and Au/Ni/Au NWs with tagging upon green light excitation of 532 nm.

Figure 4 shows the optical reflectance and fluorescence images of different Au nanostructures with or without dye-attached DNA. The results show that the NWs without DNA tagging produced a bright reflection on the NWs [22] (Figure 4a). At 540 nm light irradiation, no fluorescence was observed in the Au NWs without tagging (Figure 4b). In Figure 4c,d, Au NWs exhibit a bright reflection because of an uniform coverage of biomolecules with optimized distance on the surface of the wires [23]. In multilayer structures, as shown in Figure 4e,f, the image clearly shows the distinct optical signature of low and high optical reflectivities of Ni and Au [12]. Interestingly, the dye-attached DNA preferentially absorbed at the Au NWs which decreases the fluorescence intensity by optical quenching in Ni surface. Therefore, the fluorescence on the gold segment reflects brighter intensity than the Ni segment. This finding confirms that the samples exhibited respective emission based on Au shape and wire length. The fluorescence imaging results provide clear evidence that the Au NWs and multilayer NWs showed better reflectivity. To give further evidence for the NWs, PL measurements were carried out at an excitation wavelength of 532 nm [24] for Au NWs and multilayer NWs.

**Figure 5:**
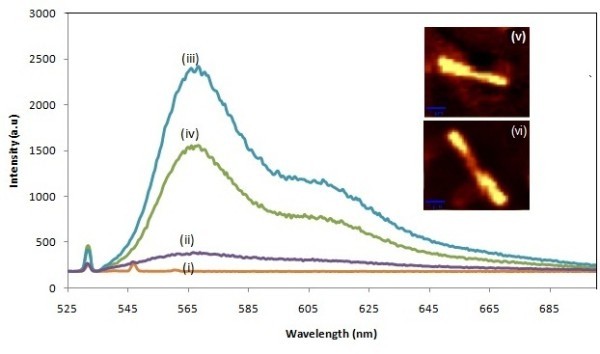
**Laser-induced PL spectra of Au NWs**. (i) Laser excitation at 532 nm on Si substrate, (ii) Au NWs without tagging, (iii) Au NWs with tagging of cy3-dye attached DNA, (iv) Au/Ni/Au NWs with tagging of cy3-dye attached DNA. PL images of (v) Au NWs and (vi) distinct difference of Au and Ni in Au/Ni/Au multilayer NWs.

Figure 5 shows the laser-induced PL emission spectrum of Au NWs and multilayer NWs. The NWs were drop-cast on a silicon substrate for PL studies. Initial measurement shows a weak emission at 542 nm, which is corresponding to the Si substrate (Figure 5i). Au NWs without tagging show a very weak and broad emission at 560 nm which is closer to gold emission [24] (Figure 5ii). Au NWs exhibited a shift of maximum emission at 570 nm, indicating the efficient tagging on the Au NWs (Figure 5iii). Figure 5iv illustrates the PL spectrum of multilayer NWs with an emission at 570 nm. The underlying concept of low emission intensity is the reduction of Au surface area in Au/Ni/Au multilayers that causes lesser amount of tagging on the NW. Consequently, a larger amount of tagged DNA is adsorbed on the Au layer, but not by the Ni layer; thus, the fluorescence signal was quenched by the Ni segment (Figure 5iv). Shown in Figure 5v,vi are the bright and dark luminescence images of the Au NW and multilayer NWs. A distinct image of Au maximum emission and Ni minimum emission was traced for the multilayer NWs, but the PL image of the Au NWs shows a complete luminescent emission from the NW surface. Therefore, a maximum emission was obtained for the Au NW when compared with the multilayer NW.

**Figure 6:**
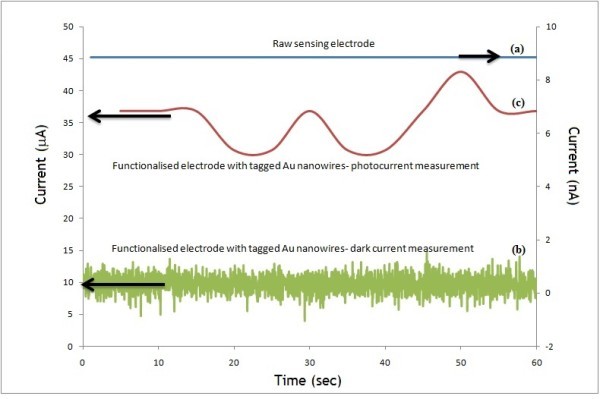
**Measurement of dark current and photocurrent from dye-attached DNA on Au NWs by PEC method**. (a) Dark current of fresh three-electrode sensor; (b) dark current measurement of functionalized electrode coated with released Au NWs, (c) photocurrent observation on functionalized electrode coated with released Au NWs.

**Figure 7:**
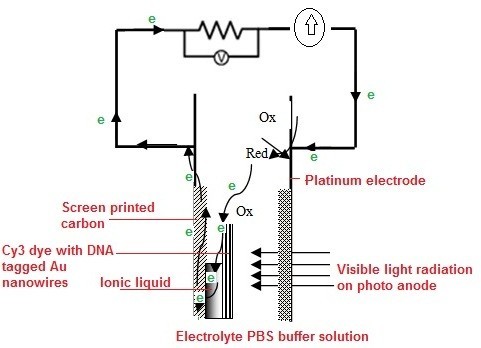
**Current mechanism of PEC behavior of Au NW coated on functionalized electrode**.

Figure 6 shows the measurement of dark and photocurrent from dye-attached DNA on Au NWs by PEC method. A dark current of 9 nA was observed for the raw electrode (Figure 6a). An improved electrical conductivity was measured for the electrodes that modified by ionic liquid with a dark current of 10 μA (Figure 6b). Under dark condition, the amount of current flow was low because there are no excited electrons in the conduction band in the dye. When the photoanode is under illumination a prominent photocurrent changed to 35 μA is observed, which is close to twofold increment in the photocurrent (Figure 6c). Figure 7 illustrates the PEC [25] behavior of the Au NW that coated on functionalized electrode. Ionic liquid helps to promote the charge transfer between the NWs and the carbon substrate. The underlying principle of the PEC behavior is the ability of photons absorption by the dye, which excites electrons to the conduction band and produces holes in the valence band that can take part in oxidation reaction. Then, the holes were driven by the internal potential of the system; where they recombine with electrons in the electrolyte. Thus, the photocurrent was generated where the reduction reaction occurred at counter electrode and oxidation reaction at photoanode. Hence, this measurement proved a simple way of diagnoses the presence of dye-attached biomolecules recognition through PEC method.

## Conclusion

In summary, Au NWs and multilayer NWs have successfully been prepared using electrodeposition technique and tagged with cy3-dye with DNA biomolecules. The optical and PEC properties have been investigated. Owing to surface plasmon resonance, Au NW showed maximum optical absorbance and PL. The PEC characteristics of Au NWs exhibited a photocurrent of 35 μA, which is because of the movement of charge carriers in the dye and their excitation to conduction band, which increase drastically the photocurrent to two orders of magnitude from initial dark current values. This study provides a platform in the area of biosensing which can be accomplished by PEC measurements.

## Competing interests

The authors declare that they have no competing interests.

## Authors' contributions

VS and HFL carried out the preparation and characterization of nanowires, participated in the sequence alignment; VS and WSL drafted the manuscript. LYH and ATP carried out the tagging of dye attached DNA into the nanowires. VS, HFL and LYH participated in the design of the study and performed the optical and fluorescence analysis. WSL conceived of the study, and participated in its design and coordination. All authors read and approved the final manuscript.
